# Arsenic-induced changes in the gene expression of lung epithelial L2 cells: implications in carcinogenesis

**DOI:** 10.1186/1471-2164-9-115

**Published:** 2008-03-03

**Authors:** Tisha Posey, Tingting Weng, Zhongming Chen, Narendranath R Chintagari, Pengcheng Wang, Nili Jin, Heidi Stricker, Lin Liu

**Affiliations:** 1 Department of Physiological Sciences, Oklahoma State University, Stillwater, OK 74078, USA

## Abstract

**Background:**

Arsenic is a carcinogen that is known to induce cell transformation and tumor formation. Although studies have been performed to examine the modulation of signaling molecules caused by arsenic exposure, the molecular mechanisms by which arsenic causes cancer are still unclear. We hypothesized that arsenic alters gene expression leading to carcinogenesis in the lung.

**Results:**

In this study, we examined global gene expression in response to 0.75 μM arsenic treatment for 1–7 days in a rat lung epithelial cell line (L2) using an in-house 10 k rat DNA microarray. One hundred thirty one genes were identified using the one-class statistical analysis of microarray (SAM) test. Of them, 33 genes had a fold change of ≥ 2 between at least two time points. These genes were then clustered into 5 groups using K-means cluster analysis based on their expression patterns. Seven selected genes, all associated with cancer, were confirmed by real-time PCR. These genes have functions directly or indirectly related to metabolism, glycolysis, cell proliferation and differentiation, and regulation of transcription.

**Conclusion:**

Our findings provide important insight for the future studies of arsenic-mediated lung cancer.

## Background

Arsenic is a carcinogen that causes lung cancer as well as skin, bladder and kidney cancers [1]. At 50 μg/liter, the cancer risk to people caused by arsenic has been estimated to be between 1300 to 1650 per 100,000 people [2]. The identification of the chemical species that are active toxicants and the mode of toxicity are both important components for accurately determining the full breadth of arsenic exposure.

Several mechanisms for arsenic-induced carcinogenesis have been proposed including genetic and epigenetic changes, inhibition of DNA repair, oxidative stress, alterations in cell death and proliferation, and aberrant activation of signal transduction pathways [3]. Exposure of TM3 testicular Leydig cells to arsenic results in the changes in DNA methylation and mutations as determined by random amplified polymorphic DNA (RAPD) [4]. Arsenic exposure reduces DNA repair probably by inhibiting DNA repair proteins such as excision repair cross-complement 1 (ERCC1) and zinc fingers DNA repair proteins [5,6]. Arsenic also alters cell-cycle related genes including cyclin D1, and cdc25A, and thus cell proliferation [7-9].

Arsenic-related gene expression studies have been performed in several different cell types [7,10,11]. Interestingly, genes involved with cellular respiration have been consistently identified in these expression studies. The addition of arsenite to human keratinocytes has been shown to lead to an increase in *thioredoxin reductase *(*TrxR*), a selenocysteine isomer involved in many cellular redox processes that is often up-regulated in cancers [12]. The same study reported that *glutathione peroxidase *(*Gpx*), which protects against reactive oxygen species (ROS), was reduced in expression upon the arsenic exposure [12]. This implies that arsenic exposure not only promotes the production of ROS but also reduces the cell's ability to defend against ROS. This is a notable concept, considering that ROS have long been known to contribute to carcinogenesis [13,14].

Arsenic activates all mitogen-activated protein kinase (MAPK) pathways, including the extracellular signal regulated protein kinase (ERK), c-Jun N-terminal kinase (JNK) and p38 kinase [15-17]. MAPK pathways are involved in cell proliferation and apoptosis. ERK activation by arsenic results in cell proliferation while JNK activation induces apoptosis. Arsenic likely activates these pathways via tyrosine kinase receptors such as the EGF receptor. In addition to activating kinases, arsenic is also known to regulate transcription factors including *AP-1 *[18-20] and *NFkB *[21-23]. These findings strongly suggest that arsenic is involved in the disturbance of the regulation of these pathways, which may lead to cancer.

We hypothesized that arsenic alters gene expression in the lung and that the alterations lead to carcinogenesis by direct and indirect means. We examined global gene expression in an arsenic-treated rat lung epithelial cell line (L2) using an in-house 10 k rat DNA microarray. The microarray data was confirmed using real-time PCR analysis of selected up- or down-regulated genes. Taken together, this study provides a valuable baseboard for the future study of arsenic-induced cell transformation.

## Results

### Cell viability after arsenic exposure

**Figure 1:**
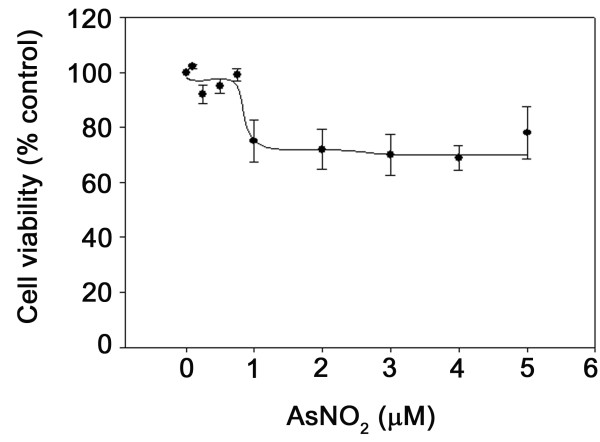
**Viability of L2 cells treated with arsenic**. L2 cells were cultured in 30 mm cell culture dishes in F12 K medium supplemented with 10% of fetal bovine serum. When grown to 80 to 90% confluence, the cells were treated for 7 days with sodium arsenite (0–5 μM). The cell viability was significantly reduced at >1 μM of arsenite as determined using MTT assay. The results were expressed as a percentage of control (untreated cells). Data shown are means ± S.E. (n = 3 cell preparations).

We first determined the viability of the L2 cells treated with arsenic in order to optimize our further experiments. When grown to 80 to 90% confluence, the cells were treated for 7 days with sodium arsenite with (0–5 μM). The cell viability was significantly reduced at >1 μM of arsenite as determined by the MTT assay (Fig. 1). Based on the results, we chose the following conditions for microarray experiments: 0.75 μM for 1, 3, 5 and 7 days.

### Microarray data analysis following arsenic exposure

**Figure 2:**
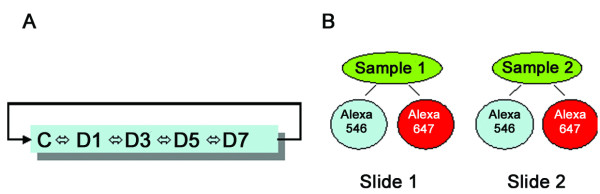
**Microarray experimental design**. A. Loop design used for microarray hybridization study. Each RNA sample was divided into 2 parts: one was labeled with Cy3 and the other with Alexa 647. Two cDNAs with different dyes were paired and hybridized to an array. B. For each paired sample, dye flip was used to overcome the difference between two fluorescent dyes because their intrinsic properties. Three cell preparations were used.

We treated L2 cells with 0.75 μM of arsenite for the assigned time periods and subsequently performed DNA microarray analysis of 10 k genes in order to determine changes in the gene expression that may be cancer-related. The samples were arranged for hybridization using a loop design (Fig. 2A). For each paired sample, dye flip was used to overcome the difference between two fluorescent dyes (Fig. 2B). There were a total of 30 hybridizations (3 cell preparations, dye flip, and 5 samples). 131 genes (100 known genes and 31 EST) passed the statistical analysis of microarray (SAM) test (P < 0.05) (see Additional file 1). Of them, 33 genes had a fold change of ≥ 2 between at least two time points. These genes would be the starting point for our study of possible carcinogenic pathways.

### Cluster analysis and major functional categories

**Figure 3:**
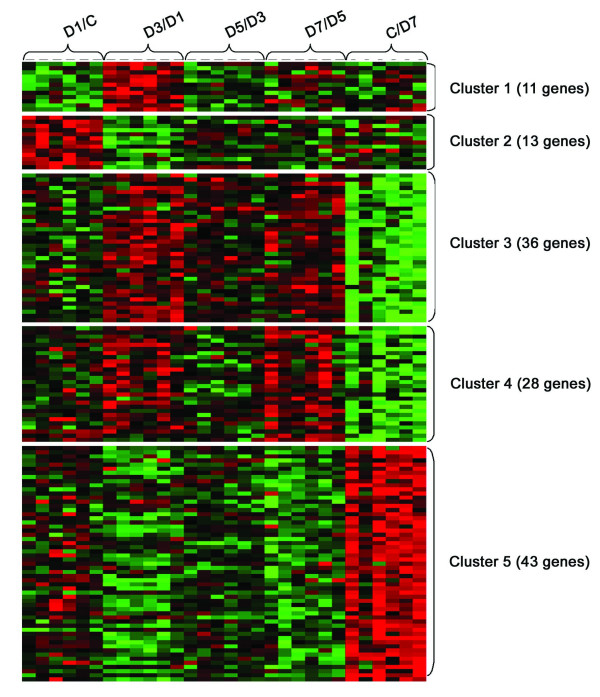
**Tree view for clustered genes**. K-mean cluster analysis of 131 genes, which were significantly changed between at least 2 time points as tested by SAM was performed using cluster and TreeView. Genes were clustered into 5 clusters. Each row corresponds to one gene, and each column corresponds to log2 ratio. Red = upregulation; green = downregulation; black = no change. Brightness of the color represents the value of the ratio. C: control untreated cells; D1-7; the cells treated with 0.75 μM for 1–7 days.

**Figure 4:**
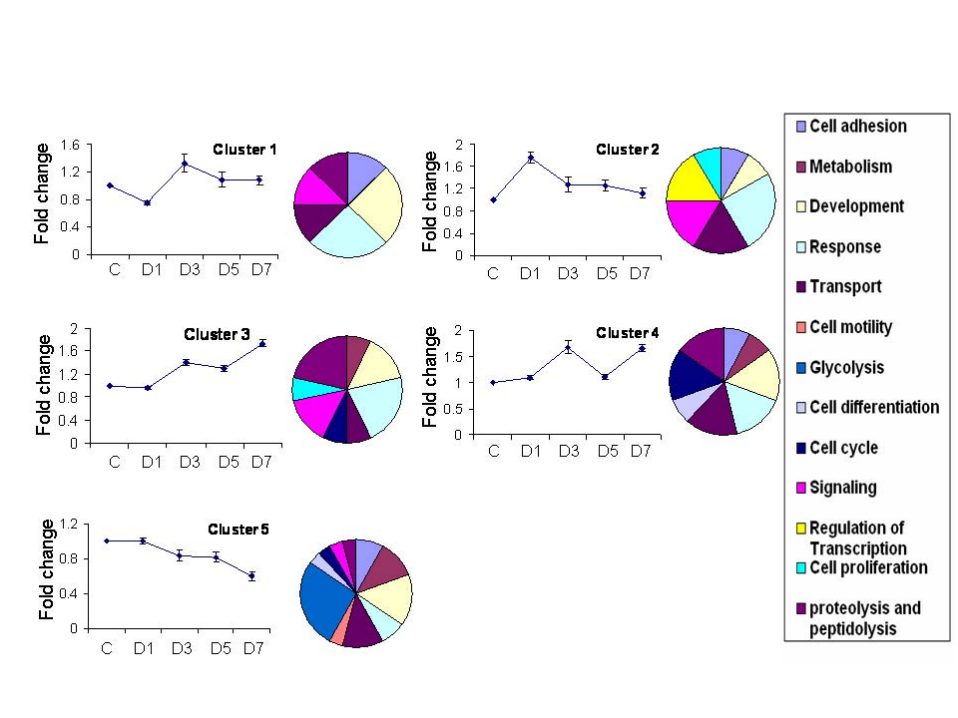
**Expression patterns and functional analysis of each cluster from microarray**. Fold change over control cells was calculated for each gene. The fold changes of all genes in each cluster identified by K-means clustering analysis were averaged and plotted as fold change v.s time. The results shown are means ± S.E. Distribution of major functional categories for biological process terms are shown in pie charts. C: control untreated cells; D1-D7; the cells treated with 0.75 μM arsenic for 1–7 days.

Following identification, we used K-means cluster analysis to cluster the 131 genes into 2–10 nodes. We found that 5 nodes or 5 clusters best represented their patterns of expression (Fig. 3). Of these genes, 78 were annotated by gene ontology [24], which described the genes in terms of their biological processes, cellular components and molecular functions in a species-independent manner. The functions of the clusters included: cell adhesion, metabolism, development, response to injury, transport, cell motility, glycolysis, cell differentiation, cell cycle, signaling, regulation of transcription, cell proliferation, proteolysis and peptidolysis (Fig. 4).

Cluster 1 (11 genes) decreased in expression on day 1 and increased on day 3 and then declined in expression on days 5 and 7. Cluster 1 is composed largely of cellular response and development. Cluster 2 (13 genes) increased in expression on day 1 and declined fairly steadily thereafter. Cluster 2 involves regulation of transcription, signaling, transport and response to cellular injury. Cluster 3 (36 genes) tended to gradually increase in expression continuously from day 0 to day 7. Cluster 4 (28 genes) had high expression on days 3 and 7. Cluster 4 is composed of a fairly even mixture of the functional categories stated. Cluster 5 (43 genes) steadily decreased in expression from day 0 to day 7. Fold changes in each cluster were lower than 2 (Fig. 4). This is because the values were the averages of all genes in each cluster. Of 11, 13, 36, 28 and 43 genes in clusters 1–5, only 4, 5, 5, 9, 10 genes in clusters 1–5 had a fold change of ≥ 2 between two time points. The temporal differences in the expression of these gene clusters provide a clue as to how these genes may participate in the process of arsenic-mediated cell toxicity.

### Real-time PCR confirmation

**Figure 5:**
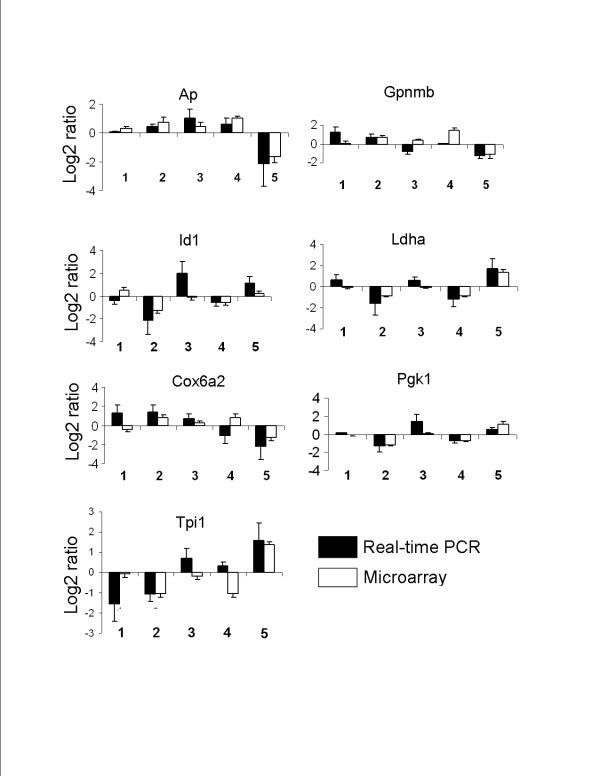
**Comparison of microarray and real-time PCR**. Total RNA was extracted from L2 cells exposed to 0.75 μM arsenic for 0, 1, 3, 5 and 7 days (C, D1, D3, D5 and D7) and reverse transcribed into cDNA. The mRNA levels were determined by real-time PCR and the data were normalized to 18S rRNA. Comparison of microarray and real-time PCR data is seen. Values are means ± SE; n = 3 cell preparations, each assayed in duplicate. D1/C (1), D3/D1 (2), D5/D3 (3), D7/D5 (4), C/D7 (5).

Based on the gene ontology analysis, we selected 11 genes with at least one gene being selected from each of the 5 clusters for real-time PCR verification. Four genes, *aldose reductase-like protein*, *matrix metalloproteinase 2 and 11*, and *alanyl membrane aminopeptidase*, had little association with the microarray and were disregarded from further investigation. For the rest of the genes, *alkaline phosphodiesterase (Ap), glycoprotein nonmetastatic melanoma protein B (Gpnmp), inhibitor of DNA binding 1 (Id1), lactate dehydrogenase A (Ldha), cytochrome c oxidase 6 a2 (Cox6a2), phosphoglycerate kinase (Pgk1), triosephosphate isomerase 1 (Tpi1)*, 69% of the log2 ratios from the real-time PCR showed similar trends with the microarray data based on log2 ratio being positive or negative even though absolute changes varied (Fig. 5). Differences in the results may be due to the difference between the two methods. Of these 7 genes, three (*Tpi1, Pgk1, and Ldha*) were from cluster 5 whose functional category is comprised largely of glycolysis. Two (*Ap and Cox6a2*) were from cluster 3 whose functional category primarily includes response, proteolysis and peptidolysis. The remaining two, *Id1 and Gpnmb *were in clusters 2 and 4, respectively. All of these genes have functions directly or indirectly related to metabolism, glycolysis and cell proliferation which may associate them with cell transformation. Table 1 shows the major functions and the cellular locations of these 7 genes.

**Table 1:** Function of genes verified via real-time PCR

Gene Name	Tissue	Major Functions	Cellular location
Id1	Mixed tissue	DNA binding; development; negative regulation of transcription from Pol II promoter; regulation of angiogenesis	Nucleus
Cox6a2	Mixed tissue	Cytochrome C oxidase activity: electron transport; oxidoreductase activity; respiratory chain complex IV	mitochondrion
Ap	Liver	Phosphodiesterase activity: catalysis of the reaction; regulate cyclic nucleotide signaling; signal transduction regulation; second messenger molecules.	Cell and nuclear membrane; subcellular domains
Ldha	Mixed tissue	L-lactate dehydrogenase activity: glycolysis; lactate dehydrogenase activity: oxidoreductase activity	Cytoplasm
Gpnmb	Bone	bone mineralization; osteoblast differentiation	Plasma membrane
Tpi1	Mixed tissue	Fatty acid biosynthesis; gluconeogenesis; glycolysis; isomerase activity; metabolism; pentose-phosphate shunt; triose-phosphate isomerase activity	Mitochondrion
Pgk1	Mixed tissue	Glycolysis; kinase activity; phosphoglycerate kinase activity; phosphorylase kinase activity; phosphorylase kinase complex; transferase activity	Mitochondrion

## Discussion and conclusion

Large scale screening of genes involved in the arsenic-induced alternation of gene expression is a practical approach for dissecting the molecular pathways of arsenic toxicity. DNA microarrays are now widely used to study differential gene expression in relation to arsenic exposure [11,25-27]. Along with our present study, these previous studies illustrate that genes identified via microarray in wide-screening of cells exposed to arsenic can be a valuable tool to determine the mechanism of arsenic toxicity and cancer formation.

In our study, we used an in-house rat DNA microarray composed of 6,221 known genes and 3,594 ESTs to investigate differential gene expression in arsenic-treated rat lung cells. This is the first study to focus on arsenic-induced gene expression alterations in the lung. Global gene expression patterns were identified by cluster analysis. Because of the notable link between arsenic toxicity and cancer formation in other organ systems, genes which have been previously linked with cancer and which may be important in arsenic toxicity were chosen from the 10 k microarray clusters to be further confirmed via real-time PCR. Real-time PCR verified 7 genes at a consistency of 69%.

One of the identified genes, *Ldha*, is involved in glycolysis in metabolism. Silencing of this gene has been shown to lead to the increased expression of the *Ldhb *gene and a subsequent abnormal expression of *lactose dehydrogenase (LD) *[28]. There seems to be a strong correlation between high *LD *expression and tumors, which has led to the use of *LD *as a marker for cancer [29,30]. Our study shows a steady decline in the expression of *Ldha *in response to arsenic, which may lead to the typical increase in *LD *seen in various cancers.

*Tpi1 *is a glycolytic enzyme found in mitochondria that catalyzes the reversible interconversion of glyceraldehyde 3-phosphate and dihydroxyacetone phosphate. It plays an important role in several metabolic pathways including fatty acid biosynthesis, gluconeogenesis and glycolysis and is essential for efficient energy production [31]. *Tpi1 *has exhibited varied expression between different types of cancer [32] and may play a role in tumor metabolism. Our study shows a steady decline in the expression of *Tpi1 *in arsenic-exposed L2 cells. Further testing will be needed to determine if this phenomenon is constant among lung cancers.

*Pgk1 *is a major enzyme found in mitrochondria. Its function involves catalyzing the reversible conversion of 1, 3-diphosphoglycerate to 3-phosphoglycerate, generating one molecule of ATP. Many studies have reported overexpression of *Pgk1 *in tumors [33-35]. However, a recent study has linked decreased *Pgk1 *expression to prostate tumor [36]. The same study also found that *Pgk1 *showed an inverse expression to CXCR4 activation, suggesting that signaling through the CXCR4 receptor regulates *Pgk1 *and vice versa. Our study showed a steady decline in arsenic-treated L2 cells. Further investigation is needed to determine the expression of *Pgk1 *in lung cancers.

*Cox6a2 *is the third gene investigated that is found in mitochondria. It is involved in cytochrome oxidase activity and is the terminal electron acceptor in the electron transport chain. Through its functions, Cox helps to produce ATP. One study found that *Cox *may be related to the progression of bladder cancer as Cox activities were found to be increased during and even after the discontinuance of carcinogen administration in rats [37]. Our study showed a steady increase in *Cox6a2*, which correlates with the increased expression seen in cancers. The location of *Tpi1*, *Pgk1 *and *Cox6a2 *suggests that mitochondrial DNA may be specifically targeted by arsenic. It has been shown that arsenic causes genetic and epigenetic changes [4]. Other studies have also linked arsenic to mitochondrial DNA damage, where mitochondrial DNA-depleted cells produced few or no mutations in response to arsenic exposure compared to intact cells [38,39]. It seems that one way arsenic confers its carcinogenicity is to simply damage components of the cellular respiratory machinery to create ROS and/or to decrease the cell's defense against ROS. The unchecked ROS is thus capable of damaging DNA to the point of inducing transformation.

Id proteins inhibit transcription by binding basic helix-loop-helix (HLH) transcription factors through their HLH motif. Id proteins are involved in cell growth, development, senescence, differentiation and angiogenesis. Several studies have linked *Id1 *to cancer [40-43]. Furthermore, Id proteins are up-regulated in cancer cells in response to hypoxia [44], which points to a secondary role in tumor maintenance as well as carcinogenesis. Our study showed an initial spike of *Id1 *expression at day 1 with a steady decline thereafter. The transient expression may reflect compensatory mechanisms in the L2 cells.

*Gpnmb *is involved in bone mineralization and osteoblast differentiation. It was found to be highly expressed in a low-metastatic human melanoma cell line [45]; however, our study showed varied expression throughout the timepoints. *Gpnmb *is a relatively novel gene and more can be determined about their functions and possible role in carcinogenesis with further studies.

In conclusion, through our gene expression profiling of the L2 cell line exposed to arsenite, we found differentially expressed genes that may be associated with cancer. Our findings show that the altered gene expression is a direct result of arsenic exposure. Our analysis links several of the identified genes with functions that include cell growth, cell differentiation and cell metabolism. Information gained from this work is valuable for the direction of future experiments involving arsenic toxicity and lung cancer formation.

## Methods

### Materials

The rat lung epithelial cell line L2 (Cat# CCL-149), a clonally isolated cell line from the terminal alveoli of adult female rats and F12 K medium were purchased from ATCC (Manassas, VA). Sodium arsenite and 3- [4,5-dimethylthiazol-2-yl]-2,5-diphenyltetrazolium bromide (MTT) was from Sigma. Fetal bovine serum (FBS), penicillin and streptomycin were from Invitrogen (Carlsbad, CA). RNase-free DNase was from Ambion Inc. (Austin, TX) and M-MLV reverse transcriptase was from Invitrogen (Carlsbad, CA). Tri Reagent was from Molecular Research Center, Inc. (Cincinnati, OH). The Pan10K rat oligonucleotide set was from MWG Biotech. Inc. (High Point, NC). SYBR Green 1 was from Qiagen (Valencia, CA). Epoxy glass slides were from CEL Associates (Houston, TX). The 3DNA array 50 kit was from Genesphere (Hatfield, PA). Microcom YM-30 columns were from Millpore (Billerica, MA).

### Cell culture and exposure

L2 cells (passages 30–40) were cultured in 30 or 100 mm cell culture dishes in F12 K medium supplemented with 10% of heat-inactivated FBS and antibiotics (1 × penicillin/streptomycin). When grown to 80 to 90% confluence, the cells were treated with 0–5 μM arsenite for 7 days or with 0.75 μM arsenite for 1–7 days. The control cells were the untreated cells at day 0. Three cell preparations (n = 3) were used for statistical analysis. At the end of incubation, the cells were used for the viability assay, DNA microarray analysis and real-time PCR analysis.

### Cell viability assay

Cell viability was determined by the MTT assay [46], which detects mitochondrial dehydrogenase activity. Cells were seeded onto 35 mm dishes and subsequently washed twice with phosphate-buffered saline. One ml of 0.5 mg/ml of MTT was then added to the dishes. After 2 h incubation at 37°C, the solution was carefully removed and 2 ml of dimethylsulfoxide added. The dishes were placed on a shaker for 10 min. The absorbance at 485 nm of the resulting solution was measured.

### RNA extraction

Total RNA was separately isolated from 3 cell preparations of the control and arsenic-treated cells using TRI REAGENT per the manufacturer's protocol (Molecular Research Ceneter, Inc, Cincinnati, OH). The cultured L2 cells in 100 mm dishes were washed three times with phosphate-buffered prior to the extraction. One ml of TRI REAGENT was added to L2 cells, followed by 0.1 ml of 1-bromo-3-chloropropane. After 2–15 min, the mixture was centrifuged at 12,000 g for 15 min at 4°C. RNA in the aqueous phase was precipitated with 0.5 ml isopropanol, washed with 1 ml of 75% ethanol, and resuspended in RNase-free water. RNA concentration was determined by a spectrophotometer (NanoDrop Technologies, Rockland, DE). RNA quality was assessed for integrity by visual evaluation of 28S and 18S rRNA bands on an agarose gel and by measurement of the absorbance at 260 nm and 280 nm.

### DNA microarray printing

We printed 10 K rat oligonucleotides (oligos) as previously described [47]. The oligos represent 6,221 known genes, 3,594 EST and 169 Arabidopsis negative controls. Each oligo was spotted in triplicate on three identical 18 × 18 mm blocks in one slide. The spot-spot distance was 160 μm and the space between blocks was 4 mm. Each block consists of 16 subarrays and can be used as an individual slide or replicate. Oligos (25 μM) were amino-modified and resuspended in 3 × SSC printing buffer, and arrayed from 26 384-well microplates onto Epoxy glass slides using an OminiGrid 100 arrayer (GeneMachine, San Carlos, CA). To immobilize oligos, the slides were incubated for 2 days at room temperature and 65% humidity, washed one time with 0.2% SDS for 2 min and then 3 times with ddH_2_O. Finally, slides were incubated in ddH_2_O for 20 min at 50°C. Printing spots were uniform as assessed by SYTO-6 staining.

### DNA microarray hybridization

There were total 3 cell preparations treated with arsenite. Each sample was arranged for hybridizations in a loop style (Fig. 2A). Dye flip was performed (Fig. 2B). Total number of hybridization for each sample was 12 (3 for cell preparations, 2 for loop-design and 2 for dye flip). There were 30 hybridizations for the whole experiment (3 cell preparations, dye flip, and 5 samples). Total RNA (10 μg) from each sample was split into two aliquots and each was reverse-transcribed into cDNA using Cy3 or Alexa Fluro 647-specific primers provided with the Genesphere kit 3DNA array 50 kit. The cDNAs were column-purified, quantified by a NanoDrop spectrophometer, adjusted to 0.5 μg/μl and mixed with 2× formamide hybridization buffer (50% formamide, 6 × SSC, 0.2% SDS) in equal volume. The paired cDNAs were then combined in a 1:1 ratio (v/v) and denatured at 80°C for 10 min. The slide was blocked with 0.1% SDS, 1% BSA and 3 × SSC at 42°C for 40 min and hybridized with labeled cDNAs at 45°C for 48 h. The slide was then washed and incubated with Cy-3 or Alexa 647-labeled dendrimers at 45°C for 3 h. After being washing, the slide was scanned using a ScanArray Express laser scanner (PerkinElmer, Boston, MA).

### DNA microarray data analysis

The scanned images were analyzed with GenePix pro 4 (Axon Instruments, Inc, Union City, CA). The raw data were subjected to quality controls using our in-house software, *RealSpot *[48]. This software automatically assigns a quality index to each spot based on fluorescence intensity and signal-to-background ratio (SBR). We set 2 as the SBR cut-off value. We filtered data with an average quality index of < 1 for all time points. Log2 ratios were then calculated from background-subtracted signal mean intensity and were normalized with the LOWESS normalization method. We then performed statistical analysis using the significant analysis of microarray (SAM) package [49]. The q-value was set at 0.05. Finally, K-mean cluster analysis was performed using Cluster and TreeView programs [50]. The microarray dataset was deposited to GEO (access number GSE 9790) [51].

### Real-time PCR

Gene verification at the mRNA level was obtained using real-time PCR on RNA samples from arsenic-treated L2 cells which were exposed for 0, 1, 3, 5 and 7 days. Total RNA was treated with RNase-free DNase to remove DNA contamination and then reverse-transcribed into cDNA using random hexamers and M-MLV reverse transcriptase. The real-time PCR primers were designed using Primer Express software (Applied Biosystems, Foster City, CA). The length of each primer was kept between the lengths of 20–25 bp with a Tm of 58–60°C (Table 2). The specificity of the primers was confirmed using the BLAST search. The real-time PCR was performed on an ABI 7500 system using a SYBR Green 1 for detection. The thermal conditions included a heat activation of the DNA polymerase at 95°C for 15 min, followed by 40 cycles of denaturing at 95°C for 20 s, annealing at 60°C for 30 s, extension at 72°C for 30 s. After the amplification, a dissociation curve was generated for each gene to check the specificity of the PCR products. The relative abundance of each gene was calculated by subtracting the C_T _value of each sample of an individual gene from the corresponding value for the 18 S gene based on the Δ Ct method [52].

**Table 2:** Real-time PCR primers of genes verified using real-time PCR

**GenBank ID**	**Gene**	**Up**	**Down**
NM_012980	MMP11	CACTTTGACTATGACGAGACTTGGA	CCTTAGCTGCTGTGGTGTGTTG
NM_012797	Id1	GAACGTTCTGCTCTACGACATGA	GCAGTATCTCCACCTTGCTCACTT
NM_012812	Cox6a2	TCGCTTAACTGCTGGATGCA	ATTGTGGAAAAGCGTGTGGTT
NM_019370	Ap	GTCTGCTGGCTGTGGTTTTACTAC	TCTTTGGTTGACTTGCTCTTCTTG
NM_017025	Ldha	GCACAAGCAGGTGGTTGACA	CATTATGCTCTCGGCCAAGTCT
AF184983	Gpnmb	ATGACCGGAACTCGTCTGATG	AATGGCAGAGTAGTTGAGGAAATGA
NM_022922	Tpi1	ACTGGAGCGACTTGCAAAGAG	TGGCATTGATAATGTCCACGAA
NM_053291	Pgk1	CAACCAGATAACGAATAACCAAAGG	CCAGGTGGCTCATAAGCACAA
AJ277957	ARL	GCCAGATGACCCTTCACTATTACA	TCTGGATGTGGAACCGAATAAGA
NM_031054	MMP2	GTTTATTTGGCGGACAGTGACA	GGCCTCATACACAGCGTCAAT
NM_031012	Anpep	CTTCTACCGCAGCGAGTACATG	TCATCGAAGCAAGGAAAGGATT

Primers were designed using Primer Express software. Each primer was 20–25 bp long with a Tm of 58–60°C. All the genes were sequences from the Rattus norvegicus. MMP11, Matrix Metalloproteinase 11; Id1, Inhibitor of DNA binding 1, helix-loop-helix protein (splice variation); Cox6a2, cytochrome c oxidase, subunit VIa, polypeptide 2; Ap, alkaline phosphodiesterase; Ldha, lactate dehydrogenase A; Gpnmb, glycoprotein nonmetastatic melanoma protein B; Tpi1, triosephosphate isomerase 1; Pgk1, phosphoglycerate kinase 1; ARL, aldose reductase-like protein; MMP2, matrix metalloproteinase 2 (72 KDa type IV collagenase); Anpep, alanyl (membrane) aminopeptidase.

## Authors' contributions

TP performed real time PCR verification and drafted the manuscript. ZW, ZC and LL participated in DNA microarray hybridization and analysis. LL, NRC, PC, and NJ participated in cell culture, viability assay and RNA extraction. HS and LL helped to draft the manuscript. LL conceived of the study, and participated in its design and coordination. All authors read and approved the final manuscript.

## Supplementary Material

Additional file 1 The genes changed in arsenic-treated rat lung epithelial L2 cells. 131 genes (100 known genes and 31 ESTs) passed the SAM test (P < 0.05). These genes were grouped into 5 clusters. Of 131 genes, 33 genes have a fold change of >= 2 between at least 2 time points (highlighted). Click here for file
